# Giant primary low‐grade fibromyxoid sarcoma arising from the left pulmonary parenchyma: A case report and literature review

**DOI:** 10.1002/cnr2.1718

**Published:** 2022-09-23

**Authors:** Reza Ershadi, Matin Vahedi, Behnaz Jahanbin, Fatemeh‐sadat Tabatabaei, Shahab Rafieian

**Affiliations:** ^1^ Department of Thoracic Surgery, Imam Khomeini Hospital Complex Tehran University of Medical Sciences Tehran Iran; ^2^ Cancer Institute, Pathology Department, Imam Khomeini Hospital Complex Tehran University of Medical Sciences Tehran Iran

**Keywords:** low‐grade fibromyxoid sarcoma, lung, pulmonary

## Abstract

**Background:**

Low‐grade fibromyxoid sarcoma is a rare painless neoplasm that primarily grows in young adults' proximal extremities and trunks. The lungs are infrequent sites for this type of sarcoma.

**Case Presentation:**

We reported a 26‐year‐old female that presented with a chief complaint of chest pain from a few months ago to Kasra hospital, Tehran, Iran, in August 2021. Chest computed tomography (CT) showed a hypodense mass with a well‐defined margin measuring 9.3 cm in the left upper lobe and multiple hypodense lesions with a lobulated appearance with a total diameter of 15.5 × 13.5 cm in the left lower lobe of the lung.

**Conclusion:**

This is the largest case of primary pulmonary low‐grade fibromyxoid sarcoma (30 × 28 × 7 cm), which seemed unresectable at first evaluation. Due to the extent of the tumor, left pneumonectomy was performed, leading to attenuation of symptoms and no recurrence at a six‐month follow‐up.

## 1 INTRODUCTION

Low‐grade fibromyxoid sarcoma is a rare painless neoplasm that primarily grows in the proximal extremities and trunk in young adults and was first described by Evans in 1987.^1^ Histologically, the tumor is characterized by slender, spindle‐shaped cells within a fibrous and myxoid area. However, the heterogeneous histological appearance makes diagnosis challenging. Immunohistological assessment could be helpful for a definitive diagnosis of low‐grade fibromyxoid sarcoma. Systemic chemotherapy or radiation are usually ineffective due to low nuclear grade and mitotic activity of low‐grade fibromyxoid sarcoma,^2^ and thus, complete resection is the standard treatment.^3^ Although the main location of the tumor is proximal extremities and trunk, it might rarely present in unusual sites, such as the retroperitoneum, abdomen, head/neck, and mediastinum.^4^ The lungs are scarce sites for this type of sarcoma, and metastasis from other areas to the lungs is more common than primary pulmonary low‐grade fibromyxoid sarcoma. Thus, systematic investigation is necessary when labeling the tumor as primary pulmonary low‐grade fibromyxoid sarcoma to rule out metastasis. A total of five cases of primary pulmonary low‐grade fibromyxoid sarcoma have been reported previously.^5^, ^6^, ^7^, ^8^, ^9^ In this study, we report a giant low‐grade fibromyxoid sarcoma arising from the left lung and occupying the whole left hemithorax. The primary distinction from previous cases is the extremely large size of the tumor and the course of surgical treatment.

## 2 CASE PRESENTATION

**FIGURE 1:**
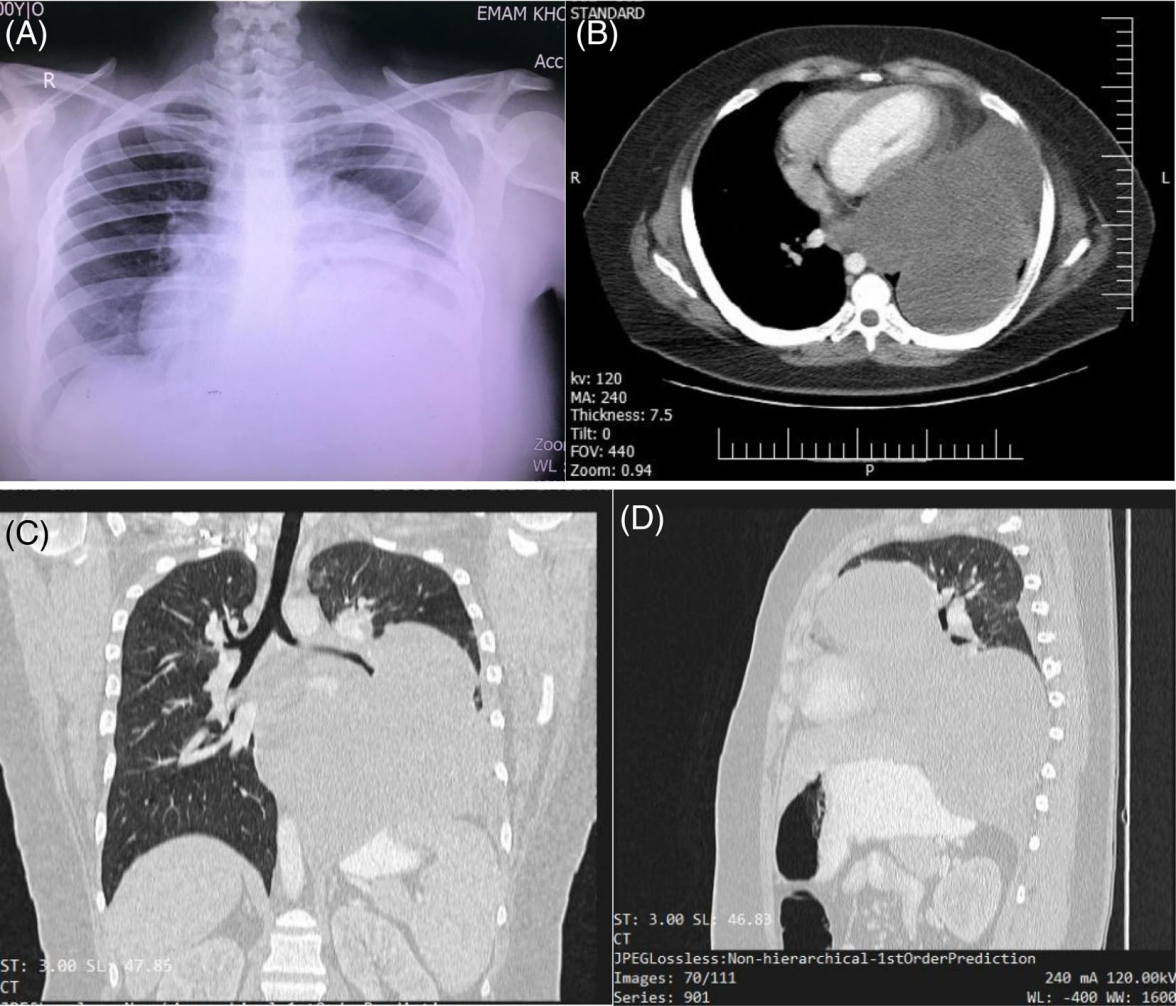
Huge lobulated mass in left hemithorax with a right‐sided shift of mediastinum. (A) chest X‐ray, (B) axial view of CT scan, (C) coronal view of CT scan, (D) sagittal view of CT scan

In August 2021, a 26‐year‐old female with unremarkable past medical history presented with a chief complaint of chest pain from a few months ago to Kasra hospital, Tehran, Iran. She had also experienced progressive shortness of breath afterward. On the first admission, physical examination showed no abnormal findings, except for decreased auscultation of the left lung. Chest X‐ray revealed a huge circular, homogenous mass lesion occupying most of the left hemithorax (Figure 1A). Follow‐up chest computed tomography (CT) showed a hypodense mass with a well‐defined margin measuring 9.3 cm in the left upper lobe and multiple hypodense lesions with a lobulated appearance with a total diameter of 15.5 × 13.5 cm in the left lower lobe of the lung (Figure 1B,C,D). In August 2021, the patient underwent transthoracic needle biopsy, which revealed hypocellular bland‐looking spindle cell proliferation in the loose edematous stroma. Immunohistochemistry results from needle biopsy showed negative staining for S100, smooth muscle actin, and CD34 and 1% staining for Ki67. No specific diagnosis was concluded. In August 2021, she underwent a thoracotomy, which was unsuccessful, and the surgeon conducted no resection. However, due to worsening symptoms, the patient was referred to our tertiary center (Thoracic surgery ward of Imam Khomeini complex hospital Tehran, Iran). In September 2021, we gave it a second chance, and a left postero‐lateral thoracotomy was carried out. A giant lobulated mass arising from pulmonary parenchyma was evident, occupying all left hemithorax. We performed left pneumonectomy. Although the tumor had some adhesions to the left pulmonary veins and arteries, the tumor had not invaded the hilar vessels. The adhesions were removed successfully with surgical intervention with no complications, and no intraoperative cardiopulmonary bypass was needed. The patient was uneventful in the postoperative period.

**FIGURE 2:**
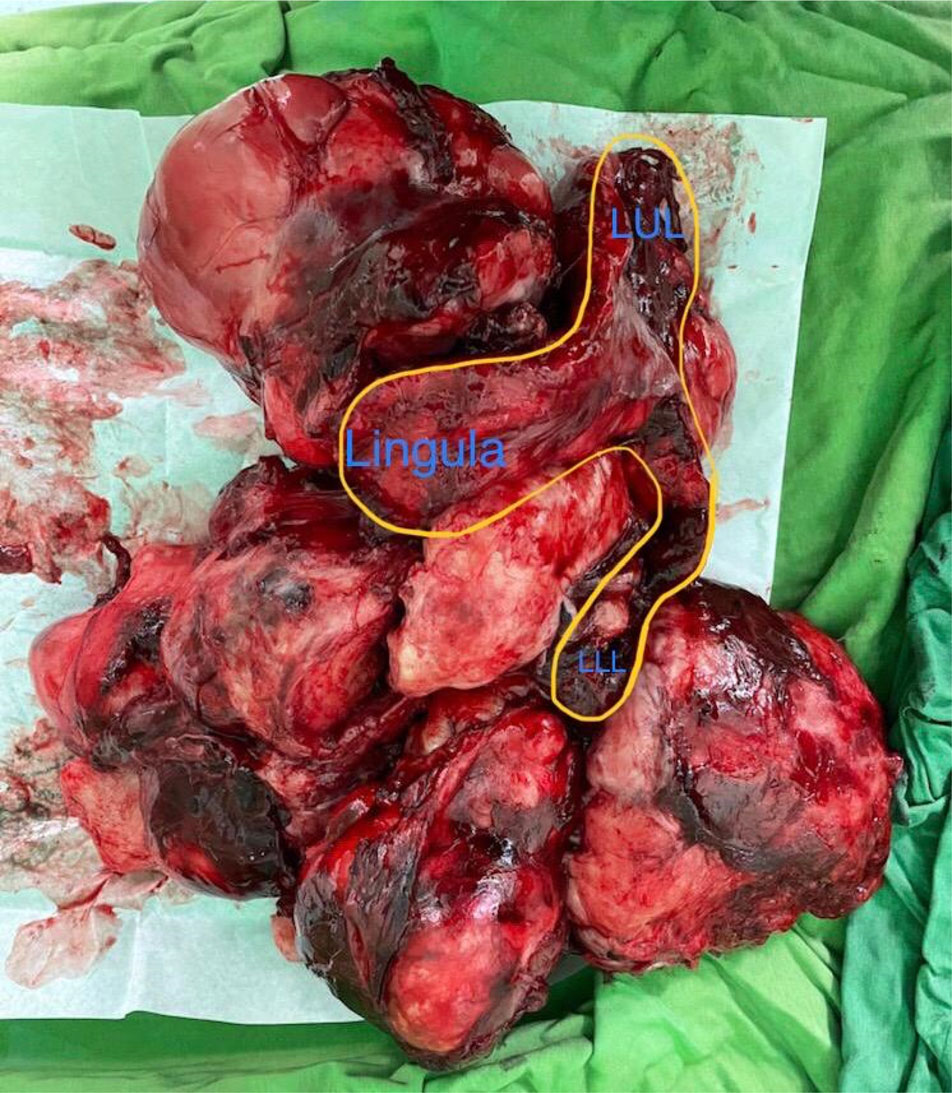
Gross view of resected tumor. LLL, Left lower lobe; LUL, Left upper lobe

**FIGURE 3:**
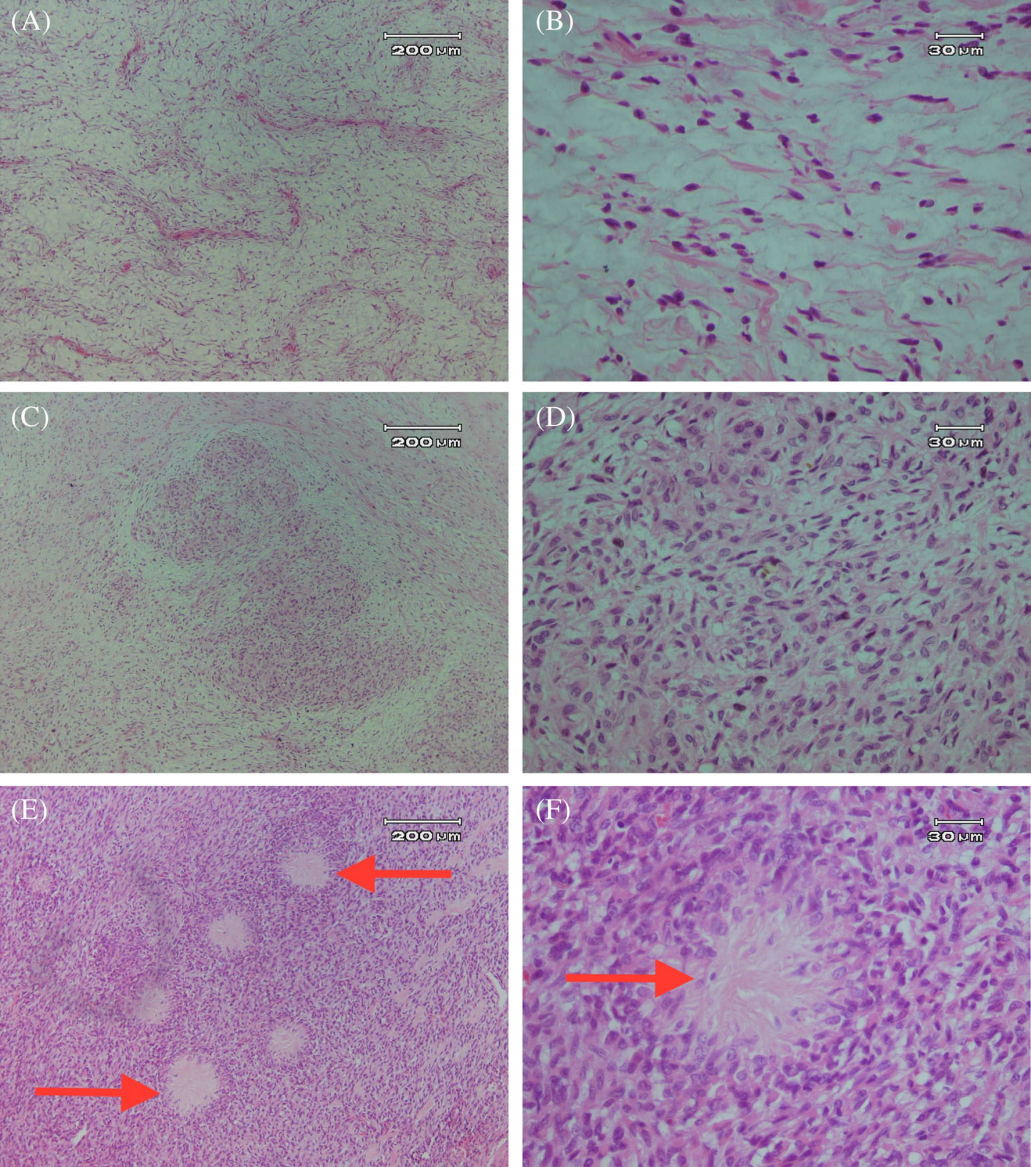
Pathological examinations of the resected tumor. (A) Hypocellular myxoid areas with curvilinear vessels (H&E staining, 10×) and (B) spindle to ovoid dark nuclei without pleomorphism in myxoid areas (40×). (C) Alternating hypo and hypercellular areas with a collection of epithelioid cells (10×), (D) with plump nuclei and acidophilic cytoplasm (40×), (E) Rosette structures in some areas (10× magnification), (F) which composed of collagen fiber in the center and collection of epitheliod cells at periphery (40× magnification). Arrows show Rosette structures

**FIGURE 4:**
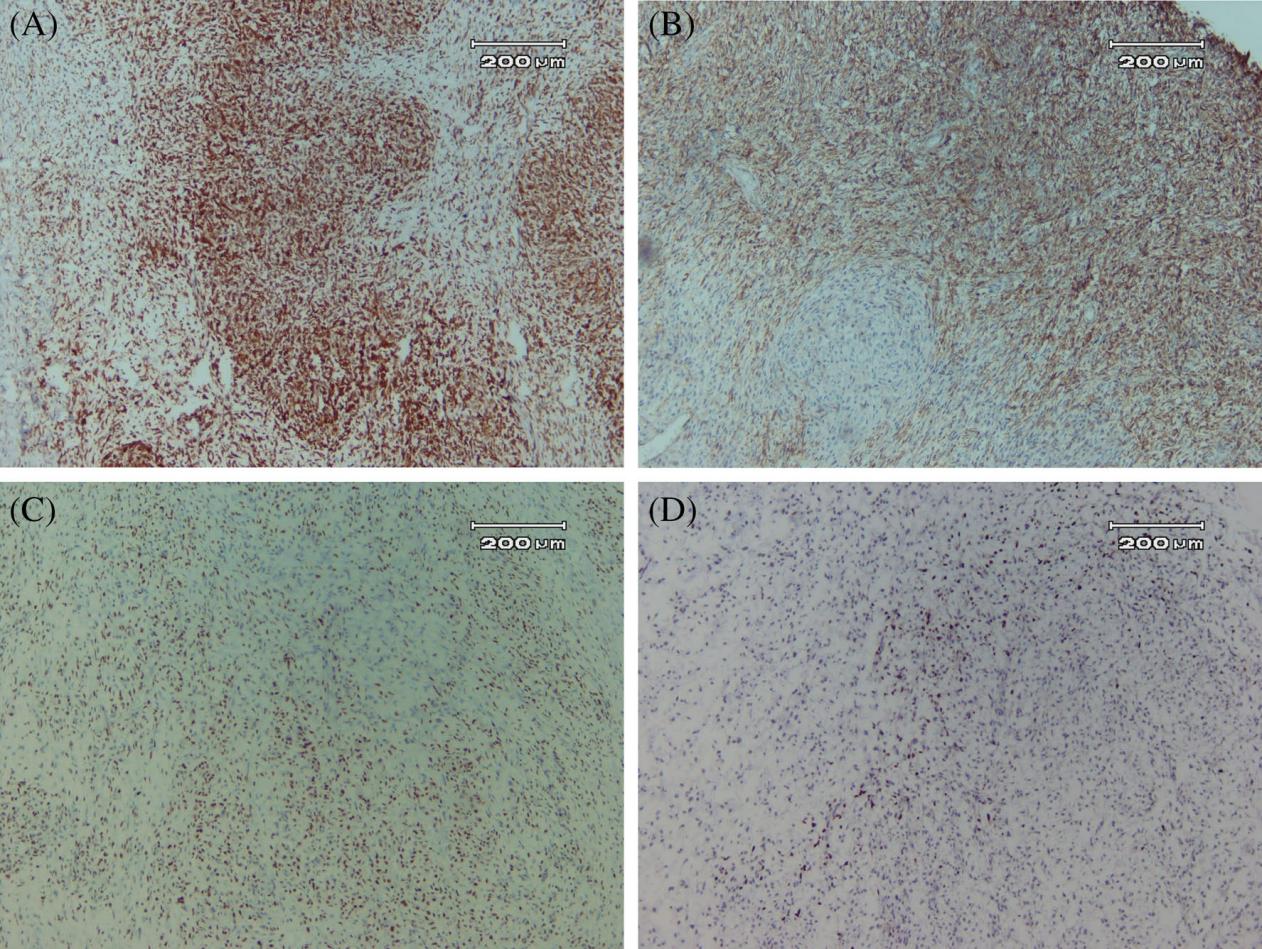
Immunohistochemistry of the resected tumor. (A) Immunostaining of Bcl2 (10×), (B) CD99 (10×), (C) focal nuclear staining for TLE1 (10×), and (D) low Ki‐67 labeling index (10×)

The pneumonectomy specimen consisted of a well‐circumscribed, homogeneous tumor measuring 30 × 28 × 7 cm with a portion of the lung measuring 10 × 8 × 4 cm and a small portion of the bronchus (1.5 × 1.2 cm) (Figure 2). Microscopically, the tumor was composed of alternating fibrous and myxoid stroma with occasional collagen rosettes. In the hypocellular area, tumor cells had dark oval spindle to ovoid nuclei without obvious pleomorphism. In some areas, a collection of epithelioid cells in a whirling pattern with plump nuclei and acidophilic cytoplasm were noted. Rosettes revealed collagen fibers in the center and a collection of epithelioid cells at the periphery. Curvilinear vessels predominantly in myxoid hypocellular areas are prominent (Figure 3). Mitotic figures were scanty, and focal areas of ischemic necrosis were noted. In the immunohistochemistry study, tumor cells were negative for CKAE1/AE3, EMA, S100, Sox10, and CD34. Patchy weak staining of BCL2, Cd99, desmin, and TLE1 with very low proliferative activity in Ki‐67 staining was also noted (Figure 4). These findings were in favor of the diagnosis of low‐grade fibromyxoid sarcoma.

At the six‐month follow‐up, the patient was disease‐free and had no symptoms.

## 3 DISCUSSION

Low‐grade fibromyxoid sarcoma is a rare neoplasm, which Evans first described in 1987 as a painless lesion of soft tissue around the scapular and axillary‐chest regions.^1^ In addition to deep soft tissues of the proximal extremities and trunk, there are several reports of this sarcoma in unusual areas. The low‐grade fibromyxoid sarcoma can originate from the extremities (45.1%), pelvis (18.6%), chest wall (13.7%), retroperitoneum (8.8%), abdomen (7.8%), and head/neck (5.9%).^4^ In a recent study, Perez et al.^10^ reviewed the cases with primary low‐grade fibromyxoid sarcoma in the intrathoracic area. Intrathoracic low‐grade fibromyxoid sarcoma involves the mediastinum, chest wall, heart, pleura, and the pulmonary parenchyma. Lungs are the main location for metastasis of low‐grade fibromyxoid sarcoma. However, the primary low‐grade fibromyxoid sarcoma of the lungs is extremely rare.

To the best of our knowledge, only five cases of primary pulmonary low‐grade fibromyxoid sarcoma have been reported.^5^, ^6^, ^7^, ^8^, ^9^ Table 1 summarizes the characteristics of these cases. Two of these were men, and three were women. The age ranged from 16 to 50 years old. Most patients presented with chest pain and shortness of breath, as in our case. However, there is also a report of an asymptomatic patient with primary pulmonary low‐grade fibromyxoid sarcoma. Left and right lungs are equally affected. The size of the previous cases ranged from 2 to 27 cm. However, our case had a larger tumor than previous ones, reaching 30 cm and occupying all left hemithorax. The results of immunohistochemistry varied between cases. All previous cases were treated with lobectomy. In our case, due to the large size of the tumor, left pneumonectomy was performed. Only two previous studies had followed up the patients and found no recurrence after 1 and 2 years.^5^, ^8^

**TABLE 1:** Description of previous cases of primary pulmonary low‐grade fibromyxoid sarcoma

Study	Age/sex	Symptoms	Side	Size/focality	IHC	Treatment	Local recurrence/follow‐up (Months)
Magro et al.^1^	20F	‐	‐	2 cm, multiple masses	‐	‐	No/12
Kim et al.^2^	50F	Chest pain	Left	7.5 cm, single mass	Positive: Vimentin, S‐100 protein and Leu7 Negative: Cytokeratin, desmin, α‐smooth muscle actin, CD34, epithelial membrane antigen, neuron‐specific enolase and calretinin	Surgery (lobectomy)	‐
Kurul et al.^3^	16F	Pain on both arms and legs	Left	10 × 8 cm, single mass	Positive: Vimentin Negative: Smooth muscle actin, muscle‐specific actin, Desmin, S‐100, Pankeratin, EMA, CD34, CD23, CD35, CD21, CD1a, CD68, ALK or Fascin	Surgery (lobectomy)	No/24
Novikov et al.^4^	41 M	Chest pain and shortness of breath	Right	27 × 22 cm, single mass	Positive: Desmin Equivocal: Smooth muscle Actin Negative: MC, S‐100, CD 34, CD‐117, and ALK‐1	Surgery (lobectomy)	‐
Yoshimura et al.^5^	22 M	Asymptomatic	Right	4 cm, single mass	Positive: bcl‐2, MUC4 Negative: EMA, AE1/AE3, CD34, STAT‐6	Surgery (lobectomy)	‐

Low‐grade fibromyxoid sarcoma is histologically characterized by spindle‐shaped cells with fibrous and myxoid areas. However, the histological findings are often heterogeneous, which makes low‐grade fibromyxoid sarcoma difficult to distinguish from other sarcomas and benign tumors.^4^ As a result, preoperative diagnosis of primary pulmonary low‐grade fibromyxoid sarcoma is controversial. In this study, we were not able to confirm the diagnosis with chromosomal translocations involving the FUS gene, and the diagnosis was confirmed after ruling out other possible diagnoses, including synovial sarcoma and carcinoma, neural tumors, myxoma, solitary fibrous tumor, and so forth, based on morphological and immunohistochemistry findings.

Comparable to other primary sarcomas of the lung,^10^, ^11^, ^12^, ^13^ the standard treatment for primary pulmonary low‐grade fibromyxoid sarcoma is complete resection since systemic therapy is associated with limited efficacy. All previous cases of primary pulmonary low‐grade fibromyxoid sarcoma have been treated with lobectomy. More recently, the National Cooperative Sarcoma Groups designed a clinical trial on combination trabectedin and radiotherapy for some types of sarcomas, including low‐grade fibromyxoid sarcoma.^13^ However, systemic treatment of low‐grade fibromyxoid sarcoma needs further investigation.

Low‐grade fibromyxoid sarcoma is associated with local or distant reoccurrence. The rate of local recurrence rate is 1%–9%, and the rate of metastasis is 6%–27% at a median follow‐up of 24–60 months.^2^, ^3^, ^4^ With regard to primary pulmonary low‐grade fibromyxoid sarcoma, only two studies reported the lack of local recurrence after 1 and 2 years of follow‐up.^5^, ^8^ However, even complete resection of low‐grade fibromyxoid sarcoma might require follow‐up for several years. In our case, the patient was disease‐free 6 months after surgery.

In conclusion, we reported a case of huge primary pulmonary low‐grade fibromyxoid sarcoma in a young adult woman. This is the largest case of primary pulmonary low‐grade fibromyxoid sarcoma, which seemed unresectable at first evaluation. Due to the extent of the tumor, left pneumonectomy was performed, leading to attenuation of symptoms and no recurrence. In addition to its size and rarity, this case highlights the surgeon's role in correct decision‐making regarding the extent of surgical intervention. Moreover, this case highlights the situation where the diagnosis is confirmed with other evidence other than specific marker immunostaining and chromosomal translocations analysis, which might not be available in all regions of the world.

## AUTHOR CONTRIBUTIONS

**Reza Ershadi:** Conceptualization (equal); investigation (equal); project administration (equal); supervision (equal); validation (equal); visualization (equal); writing – original draft (equal); writing – review and editing (equal). **Matin Vahedi:** Conceptualization (equal); investigation (equal); project administration (equal); supervision (equal); validation (equal); visualization (equal); writing – original draft (equal); writing – review and editing (equal). **Behnaz Jahanbin:** Data curation (equal); investigation (equal); validation (equal); writing – review and editing (equal). **Fatemeh‐sadat Tabatabaei:** Data curation (equal); investigation (equal); writing – review and editing (equal). **Shahab Rafieian:** Conceptualization (equal); investigation (equal); project administration (equal); resources (equal); supervision (equal); validation (equal); visualization (equal); writing – review and editing (equal).

## CONFLICT OF INTEREST

The authors have stated explicitly that there are no conflicts of interest in connection with this article.

## ETHICS STATEMENT

Investigations were in accordance with the Helsinki Declaration of 1964 and all subsequent revisions.

## INFORMED CONSENT STATEMENT

Informed consent was obtained from the patient to publish case details and use images.

## Data Availability

Data sharing is not applicable to this article as no new data were created or analyzed in this study.
